# High-protein golden sea cucumber jelly candy as a nutritional innovation to enhance dental and jaw development in children with childhood malnutrition (1–5 years): Evidence from an experimental Wistar rat model

**DOI:** 10.5455/javar.2026.m1057

**Published:** 2026-06-22

**Authors:** Noengki Prameswari, Bambang Sucahyo, Budi Handayani

**Affiliations:** 1 Department of Orthodontics, Faculty of Dentistry, Universitas Hang Tuah, Surabaya 60111, Indonesia

**Keywords:** Childhood malnutrition, golden sea cucumber, high-protein jelly, dental eruption, mandibular growth, pediatric dentistry

## Abstract

**Objectives:** Childhood malnutrition is often linked to delayed dental eruption and impaired jaw growth, which increase malocclusion risk in children. Nutritional innovations such as golden sea cucumber may offer functional benefits. This study aims to evaluate the effect of high-protein golden sea cucumber jelly candy on dental eruption and mandibular growth in a childhood malnutrition model.

**Materials and Methods:** An experimental post-test-only control group design was conducted using Wistar rats, divided into three groups: K1 (negative control, normal diet), K2 (positive control, low-protein diet), and P (treatment, low-protein diet with golden sea cucumber jelly candy supplementation). Parameters assessed included serum insulin-like growth factor-1 (IGF-1) levels, amino acid profiles, dental eruption rate, mandibular length and height, and osteogenesis markers (osteoblast count using *Runx2* expression).

**Results:** The treatment group (P) showed significant improvements compared with the childhood malnutrition control (K2). Supplementation restored mandibular length and height to levels similar to K1, significantly increased osteoblast number and *Runx2* expression, improved leucine levels and body weight, and accelerated dental eruption close to normal growth patterns.

**Conclusions:** Golden sea cucumber jelly candy has potential as a functional nutritional intervention to promote dental eruption and jaw development under childhood malnutrition conditions, supporting preventive and pediatric dentistry approaches.

## 1. Introduction

Stunting remains a critical health concern in Indonesia, where the World Health Organization (WHO) reported that the prevalence among children under five ranks third highest in Southeast Asia [1]. Stunting is defined as impaired growth and development resulting from chronic malnutrition during the critical first 1000 days of life. Stunting in children begins from the early stages of life until the first three or four years [2]. Prolonged stunting has wide-ranging implications on physical growth, cognitive development, immunity, and overall health status [3]. Within pediatric dentistry, children with childhood malnutrition often present with delayed tooth eruption and higher susceptibility to dental caries, both of which may contribute to malocclusion and long-term oral health problems [4, 5]. National data from 2013 revealed that 24% of Indonesian infants were born stunted, and by 2018, nearly 90% had developed dental caries before the age of five [6].

Previous studies in East Java demonstrated a significant association between balanced nutrition, tooth eruption, and malocclusion in stunted children [7]. A diet deficient in protein and essential amino acids disrupts growth regulation pathways, particularly the mechanistic target of rapamycin complex 1 (mTORC1), which integrates signals from nutrients, growth factors, and energy metabolism [8]. This disruption affects chondral plate development, skeletal muscle, organ size, and craniofacial growth, including the jaw and dentition. Reduced intake of protein and calories also lowers the production of insulin-like growth factor-1 (IGF-1), further impairing bone and dental development [9, 10, 11].

Golden sea cucumber (*Stichopus hermanii*), a marine bioresource, contains approximately 55% protein (dry weight), collagen, essential amino acids, flavonoids, mucopolysaccharides, omega fatty acids, vitamins, and minerals. The sea cucumber powder used in this research was characterized and contains 34.25% total protein and 24.03% collagen (Kjeldahl analysis), 5.32% flavonoids, and abundant glycine (32,446.55 ppm), together with essential minerals and bioactive fatty acids. It has been shown to enhance bone health, osteogenesis, and tissue regeneration while being safe for long-term consumption [12, 13]. Previous studies have explored sea cucumber extracts in nutraceutical forms such as jelly powder [14, 15, 16]; however, their application in pediatric functional food remains limited.

Developing a high-protein jelly candy derived from golden sea cucumber represents an innovative approach for stunted children. Jelly candy is widely accepted by children due to its palatable texture and flavor, making it a practical vehicle for nutrient delivery. Unlike conventional vitamin-enriched jelly products, protein-rich formulations targeting skeletal and dental growth are not yet available. Therefore, a preclinical study was designed to evaluate the effectiveness of golden sea cucumber-based high-protein jelly candy on tooth eruption, mandibular growth (length and height), amino acid levels, IGF-1 concentration, and osteogenesis markers such as osteoblast count and *Runx2* expression. This study focused on the primary dentition phase (1–5 years equivalent in humans), which is critical for establishing early oral and craniofacial development in stunted conditions.

## 2. Materials and Methods

### 2.1. Ethical approval

All experimental procedures involving animals were reviewed and approved by the Ethical Committee of Dentistry Faculty, Universitas Hang Tuah, Surabaya, Indonesia (Ethical Approval No.: S.Ket/095/KEPK-FKGUHT/VI/2025), and were conducted in accordance with the National Institutes of Health guidelines for the care and use of laboratory animals.

### 2.2. Materials

Wistar rats (*Rattus norvegicus*), low-protein diet pellets (5%), standard laboratory feed, golden sea cucumber (*Stichopus hermanii*) powder, carrageenan, gelatin, maltodextrin (or sorbitol), citric acid, distilled water, ketamine and acepromazine for anesthesia, buffered formalin, ethylenediaminetetraacetic acid (EDTA) for decalcification, enzyme-linked immunosorbent assay (ELISA) kits for insulin-like growth factor-1 (IGF-1) analysis, and high-performance liquid chromatography (HPLC) reagents for amino acid profiling.

### 2.3. Methods

This experimental study was conducted using 18 Wistar rats. Animals aged 10–21 days (corresponding to human children aged 1–5 years) were used. A post-test-only control group design was employed. Sample size was determined using Dahlan’s formula, with six replicates per group plus one reserve animal [7]. Randomization was performed using simple random allocation. with animals randomly allocated into three groups: group K1 (negative control): normal diet without intervention, group K2 (positive control): low-protein diet (5%); and group P (treatment): low-protein diet (5%) supplemented with high-protein golden sea cucumber (*Stichopus hermanii*) jelly candy. Wistar rats were acclimatized for one week under standard laboratory conditions with free access to food and water. Weight was monitored regularly to ensure health criteria were met prior to treatment.

The golden sea cucumber (*S. hermanii*) powder used in this study was prepared through a self-extraction process. Freeze-drying was selected because it minimizes thermal degradation and preserves protein integrity, amino acids, and bioactive compounds compared with conventional heat-drying methods. Self-extraction allowed standardization of the processing procedure and ensured consistency of the raw material throughout the study, minimizing variability in composition, and the use of freshly processed sea cucumber material was intended to preserve naturally occurring bioactive components, including proteins, collagen, and other nutritional compounds that may contribute to the biological effects observed in this study. Preparation of high-protein jelly candy: for 100 ml of jelly, 2 gm of golden sea cucumber powder, 1 gm carrageenan (gelling agent), 0.5 gm gelatin (optional), 1–2 gm maltodextrin or sorbitol, and natural flavoring (citric acid) were dissolved in 100 ml distilled water. Gelatin and carrageenan were dissolved in boiling water (1:2), followed by golden sea cucumber powder in hot water (1:1). Maltodextrin, citric acid, and sea cucumber powder were added at 70–80°C, then combined with gelatin–carrageenan solution, boiled, cooled at room temperature (25–30°C) for 1 h, refrigerated for 24 h, and molded into 1 gm candies. Prior to jelly formulation, the self-extracted golden sea cucumber powder was characterized by accredited laboratories. The powder contained 34.25% total protein, 24.03% collagen protein, 5.32% flavonoids, and abundant glycine (32,446.55 ppm), together with essential minerals and bioactive fatty acids. These characteristics supported its selection as a functional nutritional ingredient for the jelly candy formulation.

The treatment group (P) received 1 gm of jelly candy twice daily with pellet feed for 28 days. Previous studies demonstrating that mandibular bone and condylar cartilage can undergo measurable adaptive changes within approximately 4 weeks. Histomorphometric studies have shown that alterations in masticatory function and dietary consistency induce significant changes in periosteal bone formation, osteoblast activity, and condylar cartilage growth within a 4-week period after dietary modification [17]. Tooth eruption rate was assessed by daily measurement of the clinical length of the left mandibular incisor exposed above the gingival margin using a digital caliper. The eruption rate was calculated as the increase in exposed incisor length per day (mm/day) following extraction of the reference tooth. On day 29, rats were anesthetized, and 1.5 ml blood was collected via cardiac puncture for IGF-1 analysis (ELISA) and amino acid profiling (HPLC).

Animals were then euthanized by ketamine–acepromazine overdose and decapitation. Mandibles were dissected, and mandibular length was measured using a digital caliper as the linear distance from the most posterior point of the mandibular condyle to the most anterior point of the mandibular incisor alveolar region. Mandibular height was measured from the inferior border of the mandibular body to the highest point of the condylar process. All measurements were performed three times by the same examiner. Jaws were fixed in buffered formalin and decalcified with EDTA for histological and immunohistochemical evaluation of osteoblast count and *Runx2* expression. Osteoblasts were identified morphologically as cuboidal bone-lining cells located on the bone surface. Osteoblast counts were performed under a light microscope at 400x magnification in five randomly selected fields per specimen. For *Runx2* evaluation, mandibular sections were subjected to immunohistochemical staining using a commercially available anti-*Runx2* antibody (Sigma-Aldrich, USA). Positive immunoreactivity was identified as brown nuclear staining and quantified using a semi-quantitative scoring approach. Slides were examined using a light microscope at 200x magnification. *Runx2* expression was evaluated in five randomly selected microscopic fields.

Data were statistically analyzed descriptively. Normality was assessed using the Shapiro–Wilk test, and homogeneity of variance was evaluated using Levene’s test. For data that were normally distributed and homogeneous, one-way ANOVA was applied, followed by the LSD post hoc test. For data that were not normally distributed, the Kruskal–Wallis test was performed, followed by the Mann–Whitney test for pairwise comparisons. A significance level of *p* < 0.05 was considered statistically significant.

## 3. Results

**Figure 1.**
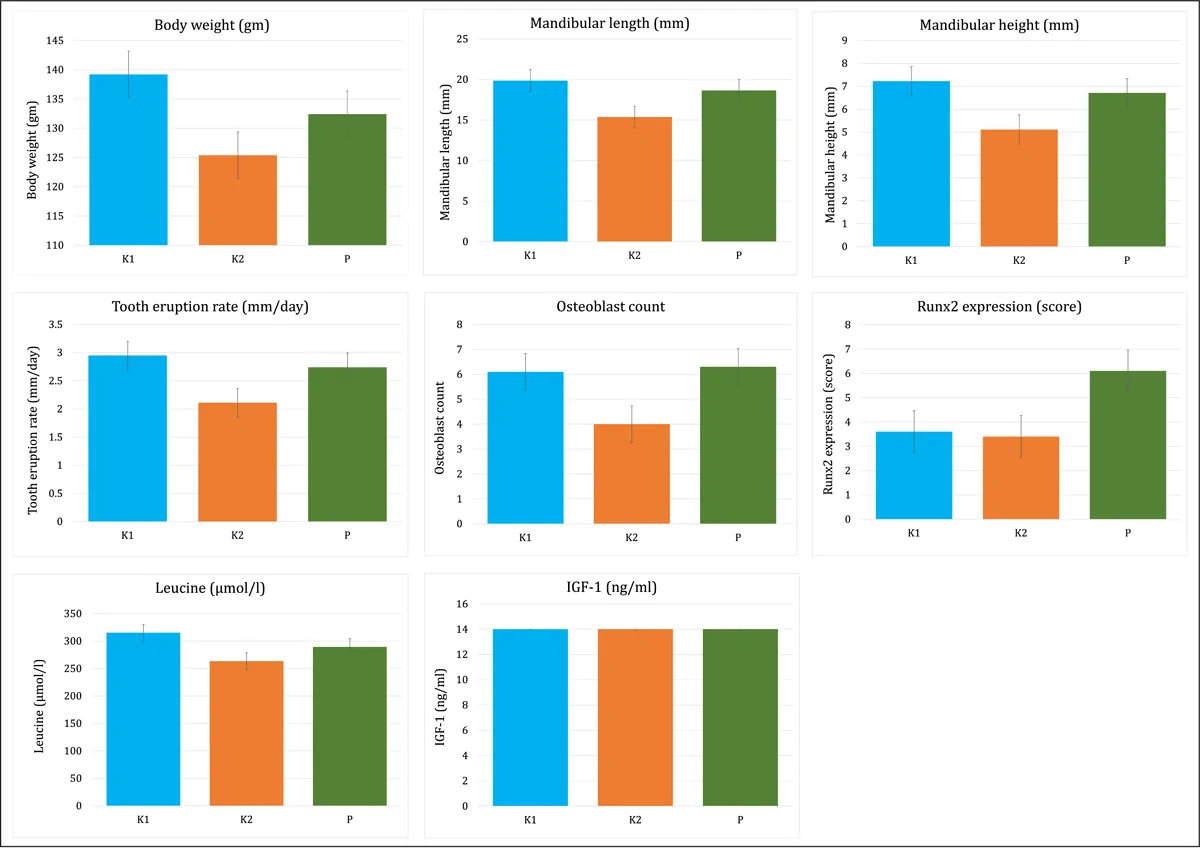
Histogram of descriptive statistics (mean) effect of high-protein golden sea cucumber jelly candy on dental eruption and mandibular growth.

Table 1 below showed that the administration of high-protein golden sea cucumber jelly candy was found to improve several mean growth-related parameters in stunted animals. Body weight in the treatment group (P) (132.4 ± 6.3 gm) increased to a level comparable with the normal control (K1) (139.2 ± 5.8 gm) and was higher than the childhood malnutrition group (K2) (125.4 ± 6.1 gm). Similarly, mandibular growth was markedly enhanced; mandibular length in the treatment group (P) (18.65 ± 1.7 mm) approached that of the normal control (K1) (19.85 ± 1.2 mm) and was considerably greater than the childhood malnutrition group (K2) (15.36 ± 1.9 mm), while mandibular height showed the same trend with values of 6.71 ± 0.9 mm in the treatment group (P) versus 7.22 ± 0.8 mm in the normal control (K1) and 5.11 ± 0.9 mm in the childhood malnutrition group (K2) (Figure 1).

**Table 1.** Descriptive statistics (mean) effect of high-protein golden sea cucumber jelly candy on dental eruption and mandibular growth.

Parameter	Groups		
	K1 (normal), *n* = 6	K2 (stunting), *n* = 6	P (treatment), *n* = 6
Body weight (gm) (mean ± SD)	139.2 ± 5.8	125.4 ± 6.1	132.4 ± 6.3
Mandibular length (mm) (mean ± SD)	19.85 ± 1.2	15.36 ± 1.9	18.65 ± 1.7
Mandibular height (mm) (mean ± SD)	7.22 ± 0.8	5.11 ± 0.9	6.71 ± 0.9
Tooth eruption rate (mm/day) (mean ± SD)	2.95 ± 0.3	2.11 ± 0.4	2.74 ± 0.2
Osteoblast count (mean ± SD)	6.1 ± 1.2	4.0 ± 1.1	6.3 ± 1.3
*Runx2* expression (score) (mean ± SD)	3.6 ± 1.0	3.4 ± 1.2	6.1 ± 1.1
Leucine (µmol/l) (mean ± SD)	315.2 ± 12.4	263.4 ± 14.6	289.3 ± 13.5
IGF-1 (ng/ml)	~14 (constant)	~14 (constant)	~14 (constant)

SD = Standard deviation.

**Figure 2.**
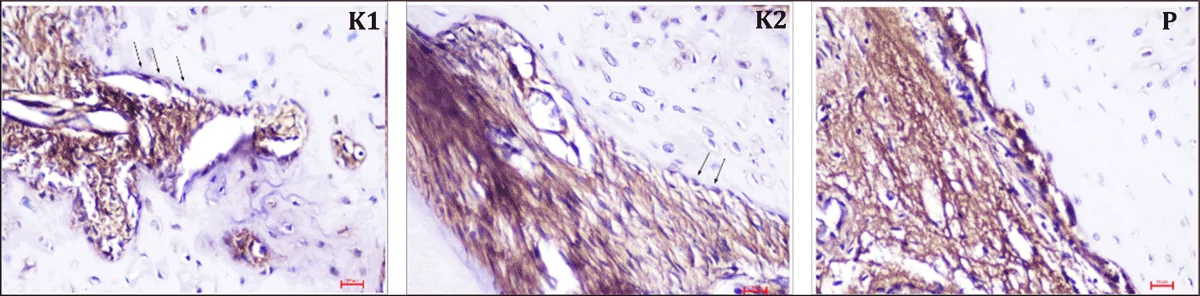
Representative images of immunohistochemical *RUNX2* expression in the mandible of Wistar rats in the normal group (K1), stunting group (K2), and golden sea cucumber treatment (P) with 200x magnification.

Tooth eruption rate in the treatment group (P) (2.74 ± 0.2 mm/day) was nearly restored to normal levels (K1) (2.95 ± 0.3 mm/day) and higher than that of the childhood malnutrition group (K2) (2.11 ± 0.4 mm/day), indicating recovery of dental development. Osteogenesis was also positively affected, as evidenced by osteoblast counts in the treatment group (P) (6.3 ± 1.3), which were greater than the childhood malnutrition group (K2) (4.0 ± 1.1) and comparable with the normal control (K1) (6.1 ± 1.2). Moreover, *Runx2* expression was remarkably elevated in the treatment group (P) (6.1 ± 1.1), surpassing both the normal (3.6 ± 1.0) and childhood malnutrition (K2) groups (3.4 ± 1.2), suggesting enhanced osteogenic activity beyond baseline levels (Figure 2).

Leucine levels also demonstrated group differences, with the highest concentration in the normal group (K1) (315.2 ± 12.4 µmol/l), followed by the treatment group (P) (289.3 ± 13.5 µmol/l), and the lowest in the childhood malnutrition group (K2) (263.4 ± 14.6 µmol/l), indicating partial but not complete restoration of amino acid profiles with supplementation. In contrast, IGF-1 levels remained constant at approximately 14 ng/ml across all groups, showing no variation and suggesting that this parameter was not sensitive to the intervention (Figure 1).

The normality test was performed using the Shapiro–Wilk method. The results indicated that the data for mandibular length, *Runx2* expression, and leucine level were normally distributed (*p* > 0.05). These parameters were therefore analyzed using one-way ANOVA. The ANOVA revealed a statistically significant difference among the groups for mandibular length (*p* < 0.05, *p* = 0.00), *Runx2* expression (*p* = 0.00), and leucine level (*p* = 0.00). Post hoc pairwise comparisons using the Tukey HSD test demonstrated that the treatment group (P) differed significantly from K2, while no significant difference was observed between K1 and P for some parameters (Table 2).

**Table 2.** Post hoc Tukey HSD test of mandibular length, leucine level, and *Runx2* expression.

Parameter	Comparison		
	K1 (normal) vs. K2 (malnutrition)	K1 (normal) vs. P (treatment)	K2 (malnutrition) vs. P (treatment)
Mandibular length	0.00*	0.846	0.00*
Leusin level	0.00*	0.00*	0.00*
*Runx2* expression	0.9	0.00*	0.00*

*indicates statistically significant.

In contrast, the parameters body weight, mandibular height, dental eruption rate, and osteoblast cell count did not meet the assumption of normality (*p* < 0.05). Accordingly, these variables were analyzed using the non-parametric Kruskal–Wallis test. The analysis showed significant group differences for body weight, *p* = 0.01, mandibular height *p* = 0.01, dental eruption rate *p* = 0.00, and osteoblast cell count, *p* = 0.00. Further Mann–Whitney post hoc tests revealed that the treatment group (P) exhibited significantly higher values compared to K2, while differences between K1 and P were not statistically significant (Table 3).

**Table 3.** Post hoc Mann-Whitney test of mandibular height, dental eruption rate, and osteoblast cell count.

Parameter	Comparison		
	K1 (normal) vs. K2 (malnutrition)	K1 (normal) vs. P (treatment)	K2 (malnutrition) vs. P (treatment)
Mandibular height	0.01*	0.62	0.02*
Dental eruption rate	0.00*	0.86	0.00*
Osteoblast cell count	0.05*	0.00*	0.04*

*indicates statistically significant.

## 4. Discussion

The study investigated the effects of golden sea cucumber (*Stichopus hermanii*) jelly candy supplementation on body weight recovery, mandibular growth, osteoblast activity, *Runx2* expression, leucine concentration, and tooth eruption in a Wistar rat model of childhood malnutrition. Characterization of the golden sea cucumber powder demonstrated substantial total protein content (34.25%) and collagen protein content (24.03%), together with the presence of several amino acids and bioactive compounds. These nutritional characteristics may have contributed to the overall improvement in amino acid metabolism observed in the treatment group. Overall, supplementation improved body weight, mandibular dimensions, osteoblast count, *Runx2* expression, serum leucine levels, and tooth eruption rate compared with the childhood malnutrition control group.

Body weight was significantly higher in the treatment group than in the childhood malnutrition group and approached values observed in the normal control group. This finding suggests that golden sea cucumber supplementation contributed to improved nutritional recovery near normal levels. Previous studies have demonstrated that marine-derived protein sources can enhance somatic growth and nutritional rehabilitation by providing proteins, amino acids, collagen peptides, and bioactive compounds that support tissue accretion and protein synthesis. The high protein and collagen content of the self-extracted golden sea cucumber powder may have contributed to efficient nutrient absorption and utilization, resulting in the observed improvement in growth performance [18]. Nutritional rehabilitation in childhood malnutrition often requires not only macronutrients but also bioactive compounds that optimize gastrointestinal function, reduce systemic energy loss from inflammation or stress, and redirect energy toward tissue accretion. Improvement in body weight observed in this study may also reflect enhanced osteoblast activity and osteogenic gene expression, since skeletal growth requires adequate protein and amino acid availability [19]. Moreover, recovery of body mass in young animals during critical growth phases increases the likelihood of compensatory catch-up growth in bone and craniofacial structures. Importantly, these findings may provide a basis for developing functional food strategies to support childhood malnutrition interventions and improve long-term growth outcomes in children.

Mandibular length and height were significantly reduced in the childhood malnutrition group, confirming that protein deficiency adversely affects craniofacial development. In contrast, supplementation with golden sea cucumber jelly candy restored mandibular dimensions to levels comparable with those of the normal control group. These findings are consistent with previous studies demonstrating that adequate nutritional intake is essential for skeletal growth, matrix formation, and mineralization. Because the experimental animals were in a phase of rapid postnatal craniofacial development, improved nutritional status may have facilitated compensatory growth of the mandible during the intervention period [20]. Dietary supplementation can stimulate mandibular endochondral growth. Myo-inositol supplementation accelerated mandibular endochondral growth in rabbits, highlighting the responsiveness of mandibular development to targeted nutritional interventions [21].

To explore the cellular basis of these skeletal changes, osteoblast activity was evaluated. The treatment group exhibited significantly higher osteoblast counts than both control groups, indicating enhanced osteogenic activity. Osteoblasts are responsible for bone matrix deposition and mineralization, and their activity is strongly influenced by nutritional status. The increase observed in the treatment group may be associated with the protein, collagen, amino acids, and other bioactive constituents present in *S. hermanii*, which have been reported to support osteoblast proliferation and differentiation.

Consistent with the osteoblast findings, *Runx2* expression was significantly elevated in the treatment group. *Runx2* is a key transcription factor regulating osteoblast differentiation and skeletal development. Adequate nutrient supply supports activation of the mTORC1 pathway, a central regulator of skeletal growth, and enhances osteoblast differentiation through the *Runx2* signaling cascade [8, 22, 23]. Increased *Runx2* expression suggests activation of osteogenic pathways during nutritional recovery. Interestingly, *Runx2* expression exceeded that observed in the normal control group. This finding may reflect an active compensatory remodeling response following protein deficiency rather than simply indicating superior bone formation. Because *Runx2* is an early osteogenic marker, elevated expression may represent ongoing osteoblast differentiation and skeletal adaptation during recovery. The bioactive composition of *S. hermanii*, which contains essential amino acids, collagen peptides, glycosaminoglycans, and micronutrients, is known to stimulate proliferation and differentiation of osteogenic precursors and osteogenic transcription factors such as *Runx2* and bone morphogenetic protein-2 (BMP-2), both of which are pivotal in initiating osteoblast differentiation [24] and also altering craniofacial proportions [25]. Additionally, improved nutritional intake is associated with enhanced systemic growth factors such as growth hormone, which synergistically support bone formation [26].

It is also important to consider biomechanical influences. Functional stimulation through adequate mastication is known to promote bone remodeling via mechanotransduction, which activates osteoblasts and regulates mandibular bone mass [27]. Although this study did not assess masticatory load directly, the provision of jelly candy may indirectly contribute to jaw stimulation. Overall, the significant increase in osteoblasts in the treatment group indicates that golden sea cucumber jelly not only prevents bone loss but actively promotes new bone formation. This highlights its potential as a functional nutritional intervention for structural and functional recovery of the mandibular bone in childhood malnutrition conditions. Adequate nutritional supply, particularly proteins, amino acids, zinc, and growth factors, directly influences signaling cascades such as BMP-2/Smad and Wnt/β-catenin, which regulate *Runx2* transcription. Zinc deficiency reduces *Runx2* expression via the BMP-2/Smad pathway in osteoblasts, highlighting the sensitivity of *Runx2* to micronutrient availability [28]. Furthermore, metabolic imbalance caused by hyperglycemia or high-fat diets has been reported to suppress *Runx2* expression and impair bone regeneration [29]. To further validate these cellular findings at the molecular level, analysis of osteogenic gene expression was performed, focusing on *Runx2*; nevertheless, interpretation should be made cautiously because additional osteogenic markers, such as BMP-2, osteocalcin, and alkaline phosphatase, were not evaluated.

Serum leucine concentrations were also significantly higher in the treatment group than in the childhood malnutrition group, suggesting improved amino acid availability and nutritional status. Leucine is an important branched-chain amino acid involved in protein metabolism and cellular growth [30]. Leucine and arginine play critical roles in activating the mTORC1 pathway and promoting osteoblastogenesis [23]. Leucine has been shown to stimulate osteoblast proliferation, collagen matrix production, and osteogenic gene expression, including *Runx2* [31]. Another study reported that leucine supplementation enhanced bone density in experimental models [32]. However, the present study did not directly quantify leucine in either the sea cucumber powder or the final jelly candy formulation. Therefore, the observed increase in serum leucine cannot be attributed exclusively to leucine supplied by the supplement. Rather, the findings likely reflect improved protein intake and metabolic recovery associated with nutritional rehabilitation. Future studies should include comprehensive amino acid profiling of both the raw material and the final formulation to clarify the contribution of specific amino acids to the observed biological effects.

The tooth eruption rate was significantly lower in the childhood malnutrition group than in both the treatment and normal control groups. This finding is consistent with previous evidence that nutritional deficiency can delay dental development through impaired cellular proliferation, matrix synthesis, and mineralization. Supplementation with golden sea cucumber jelly candy restored tooth eruption to near-normal levels, suggesting that improved nutritional status supported normal dental development and craniofacial growth during the intervention period.

Although IGF-1 was selected as a growth-related biomarker, measurable differences among groups were not detected. This finding is consistent with previous reports showing that IGF-1 levels are markedly reduced or even undetectable under conditions of malnutrition and growth impairment, limiting its diagnostic utility in such contexts [33]. Conventional immunoassays face technical limitations at very low concentrations, and advanced approaches may offer greater sensitivity for future studies [34]. Therefore, the observed improvements in mandibular growth, osteoblast count, *Runx2* expression, and tooth eruption cannot be attributed directly to IGF-1 signaling based on the present data. Alternative mechanisms related to improved nutritional status, amino acid availability, and local osteogenic responses may have contributed to the observed effects.

Several limitations should be acknowledged. First, the low-protein diet model used in this study represents childhood protein-energy malnutrition rather than the full biological complexity of human stunting, which is influenced by prenatal, environmental, infectious, and socioeconomic factors. Second, only a single concentration of golden sea cucumber powder was evaluated, preventing assessment of dose–response relationships. Third, amino acid characterization of the final jelly candy formulation was not performed, limiting interpretation of the leucine findings. Finally, only IGF-1 was assessed as a systemic biomarker, whereas additional inflammatory, osteogenic, and growth-related mediators could provide a more comprehensive understanding of the underlying mechanisms.

Despite these limitations, the present findings demonstrate that golden sea cucumber jelly candy supplementation improved nutritional recovery, mandibular growth, osteoblast activity, *Runx2* expression, and tooth eruption in a Wistar rat model of childhood malnutrition. These results provide preclinical evidence supporting further investigation of golden sea cucumber as a functional nutritional supplement for malnutrition-associated craniofacial growth impairment.

## 5. Conclusions

Supplementation with golden sea cucumber-based high-protein jelly candy effectively preserved mandibular length in a malnutrition model, demonstrating the potential of marine-derived functional nutrition to support craniofacial development. Golden sea cucumber jelly supplementation significantly enhanced *Runx2* expression, demonstrating a molecular-level mechanism by which functional nutrition can stimulate osteoblast differentiation and bone formation in childhood malnutrition conditions in an experimental Wistar rat model. Golden sea cucumber jelly supplementation was associated with higher serum leucine concentrations compared with the childhood malnutrition group, suggesting improved amino acid status and nutritional recovery. However, the specific contribution of sea cucumber-derived leucine could not be determined because amino acid characterization of the formulation was not performed.

## Data Availability

The data presented in this study are available from the corresponding author upon reasonable request.

## List of abbreviations

ANOVA, analysis of variance; BMP-2, bone morphogenetic protein-2; BCAA, branched-chain amino acid; EDTA, ethylenediaminetetraacetic acid; ELISA, enzyme-linked immunosorbent assay; HPLC, high-performance liquid chromatography; IGF-1, insulin-like growth factor-1; K1, negative control group (normal diet); K2, positive control group (low-protein diet); LSD, least significant difference; mTORC1, mechanistic target of rapamycin complex 1; P, treatment group (low-protein diet with golden sea cucumber jelly candy); *Runx2*, runt-related transcription factor 2; SD, standard deviation; WHO, World Health Organization.
